# Using Emerging Telehealth Technology as a Future Model in Vietnam During the COVID-19 Pandemic: Practical Experience From Phutho General Hospital

**DOI:** 10.2196/27968

**Published:** 2021-06-22

**Authors:** Ngoc Huy Nguyen, An Quang Nguyen, Van Thi Bich Ha, Phuong Xuan Duong, Thong Van Nguyen

**Affiliations:** 1 Department of Health Phutho Province Viet Tri Vietnam; 2 Phutho General Hospital Viet Tri Vietnam; 3 Vietnam Association of Stroke Hanoi Vietnam

**Keywords:** telehealth, telemedicine, teleconsultation, COVID-19, Vietnam, digital health, pandemic

## Abstract

Telehealth has emerged as a model of modern technology for health care services in Vietnam during the COVID-19 pandemic. To actively prevent the outbreak of COVID-19 by using a national digital transformation program, the Vietnamese Ministry of Health launched project 2628/Quyet dinh-Bo y te, which approved a scheme for remote medical examinations and treatments for 2020 to 2025. The project aims to connect 1000 hospitals to strengthen the quality of medical services by using the expertise of central hospitals to support rural areas via provincial hospitals. Phutho General Hospital (PGH) is one of leading provincial hospitals that participated in and applied the early telehealth systems in Vietnam. By using telehealth systems, PGH can offer valuable support to doctors’ activities by streamlining and facilitating their work. Telehealth was demonstrated to be feasible, acceptable, and effective at PGH in Vietnam, and it resulted in considerable improvements in health care outcomes. The COVID-19 pandemic has facilitated the acceleration and enhancement of telehealth in Vietnam. The success of telehealth in Phutho may be a useful reference for other parts of the world. However, this telehealth system focuses on the connectivity among doctors rather than the connectivity between doctors and patients, which is an area that needs further assessment.

## Introduction

Since the outbreak of COVID-19, which originated in Wuhan, China, in 2020, the disease has spread widely across the world [1,2]. The disease has affected 221 countries, and the latest data show that the outbreak has affected over 106 million people and has resulted in over 2.3 million deaths [3,4]. The primary symptoms of COVID-19 include fever, dry cough, and breathing difficulty [5,6]. Older adults and those with underlying medical problems such as hypertension, heart disease problems, and diabetes are more susceptible to developing the most severe form of the disease [7]. COVID-19 has impacted both health services and the global economy [8]. The COVID-19 outbreak has diminished prospects of an economic recovery, and many key sectors have been affected, particularly travel and tourism, retail, and other service sectors [8,9].

A method for controlling the transmission of SARS-CoV-2 is social distancing, which is made possible by the reduction of person-to-person contact [10,11]. In the context of the ongoing COVID-19 pandemic, telehealth has emerged as an ideal method for facilitating communication among people and has played a critical role in supporting the diagnosis and treatment of diseases in many hospitals [12]. Vietnam is a country that has controlled the pandemic very effectively—according to an assessment by the World Health Organization—thanks to political systems as well as the active application of new and advanced technology [13]. In the war against the COVID-19 pandemic, the Vietnamese Ministry of Health launched project 2628/Quyet dinh-Bo y te (QD-BYT) on June 22, 2020, which approved a scheme for remote medical examinations and treatments for 2020 to 2025 [14]. The National Steering Committee established the Vietnam Telemedicine Center for COVID-19 Outbreak Control in June 2020. The center frequently holds web-based consultations that involve the participation of leading professors across the country to provide advice on critical cases, discuss optimal treatments, and share experiences of inpatient treatment and care with participating experts and hospitals as if there were no distance between North and South Vietnam or high and low levels of health care. Such web-based consultations have greatly contributed to the treatment of patients with COVID-19; as of June 15, 2020, there have been no COVID-19 cases. The establishment of the Vietnam Telemedicine Center for COVID-19 Outbreak Control marked the development of medical examinations and treatment systems that are based on scientific and technological advances, especially those for dangerous infectious diseases such as COVID-19.

This paper aims to present the results of applying telehealth in Vietnam at Phutho General Hospital (PGH), which is the largest provincial hospital in the northwest region of Vietnam, as well as the advantages and challenges of early-stage telehealth in Vietnam.

## PGH’s Experience in Developing a Model for the Application of Telehealth in Vietnam

PGH, the largest public hospital in the northwest region of Vietnam, has over 1500 beds, 20 departments, and 9 centers. Currently, PGH is a satellite hospital that consists of 8 national hospitals. Hospital facilities and equipment are being enhanced, and many advanced medical technologies and techniques are being applied to medical examinations and treatments. Recognizing the importance of advancements in technology, PGH implemented telehealth technologies, which has allowed the hospital to connect with national hospitals via information technology systems. PGH has registered and signed agreements with 8 national hospitals (Table 1) as part of project 1628/QD-BYT to launch official telehealth models for medical examination and treatment services for 2020 to 2025, including teleconsultations, telesurgery consultations, telemedicine, and videoconferences.

**Table 1:** Application of telehealth at Phutho General Hospital and national hospitals.

Model	Hospital network	Achievements	Challenges	Launch year
Teleconsultation	Bach Mai Hospital, Viet Duc University Hospital, National Hospital of Tropical Diseases, and Vietnam National Cancer Hospital	Many cases on resuscitation, emergency and intensive care, surgery, respiratory diseases, oncology, etc Teleconsultations became a useful routine for the hospital network. The information technology system is relatively complete. Improving the knowledge, qualifications, and abilities of physicians Learning by doing via real cases A huge amount of recorded data for training	Information technology supporting staff is not always available Investing into the information technology systems among hospital networks Time differences and daily work among hospital networks Unavailable payment insurance Patients’ privacy Internet speed	2015
Heart surgery telementoring	Hanoi Heart Hospital	10 heart operations	Only performing simple heart surgery cases Patients’ privacy Internet speed	2020
Telemedicine	Hanoi Medical University Hospital	Daily support for patients who need the service, including patients with neurological disease, hypertension, diabetes, etc	Only cooperating with Hanoi Medical University Hospital Unavailable payment insurance Patients’ privacy	2020
Videoconference	Vietnamese Ministry of Health	Monthly meetings for direction and management	Internet speed	2020

## Implementing Telehealth to Improve Knowledge and the Quality of Treatment and Education

On June 22, 2020, given the complex issues resulting from the COVID-19 pandemic, the Vietnamese government comprehensively reviewed its epidemic prevention plan and decided to approve a scheme for “remote medical examination and treatment for 2020 - 2025.” The scheme has rapidly received strong support from the medical community, doctors, and citizens. After nearly 3 months of preparation, on September 24, 2020, the Ministry of Health of Vietnam officially launched the remote medical examination and treatment program, thereby connecting 1000 hospitals, including 20 central hospitals. The project aims to reduce the burden on central hospitals, increase the quality of medical examination and treatment in primary health care facilities, save costs, and improve patients’ experiences and satisfaction while ensuring the safety of medical staff, doctors, and patients during the COVID-19 pandemic. The program receives financial support from the Vietnamese Government for both inpatients and outpatients, regardless of whether patients have insurance or not, and telehealth services are provided free of charge. The medical specialties include the cardiology, oncology, respiratory and musculoskeletal fields.

As one of the earliest hospitals to participate in the project, PGH launched a telehealth clinic on November 14, 2020. Teleconsultations were one of the applications that physicians in PGH used commonly to consult with specialists from Bach Mai hospital, Viet Duc Hospital, the National Hospital of Tropical Diseases, and the Vietnam National Cancer Hospital (Table 1). The teleclinic office is equipped with a 52-inch screen, a 48-inch screen, and 2 computers with a high-speed internet connection. Doctors in PGH have to prepare PowerPoint presentations about patients in advance to present cases to and discuss them with specialists (ie, cases that need help and expertise). Teleconsultations helped PGH improve their medical staff’s knowledge, improve the quality of treatment via treatment plans, ensure that appropriate referrals or evacuations were conducted, improve the accuracy of diagnoses, and provide opportunities for education. The telehealth network was high in quality and resulted in faster decision making, shorter diagnosis times, faster and better patient management, shorter lengths of hospitalization and intensive care unit stays, improved diagnostic accuracy in triage, reduced anxiety, better education, increased confidence, and fewer unnecessary procedures.

**Figure 1:**
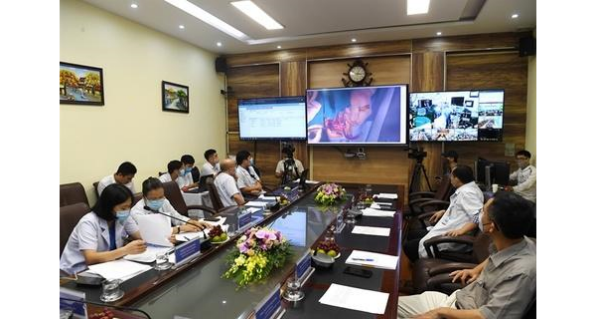
Real-time heart surgery telementoring for a case involving a 55-month-old child with a ventricular septal defect hole under 2 aortas. Telementoring was conducted during the COVID-19 outbreak between Phutho General Hospital and Hanoi Heart Hospital.

**Figure 2:**
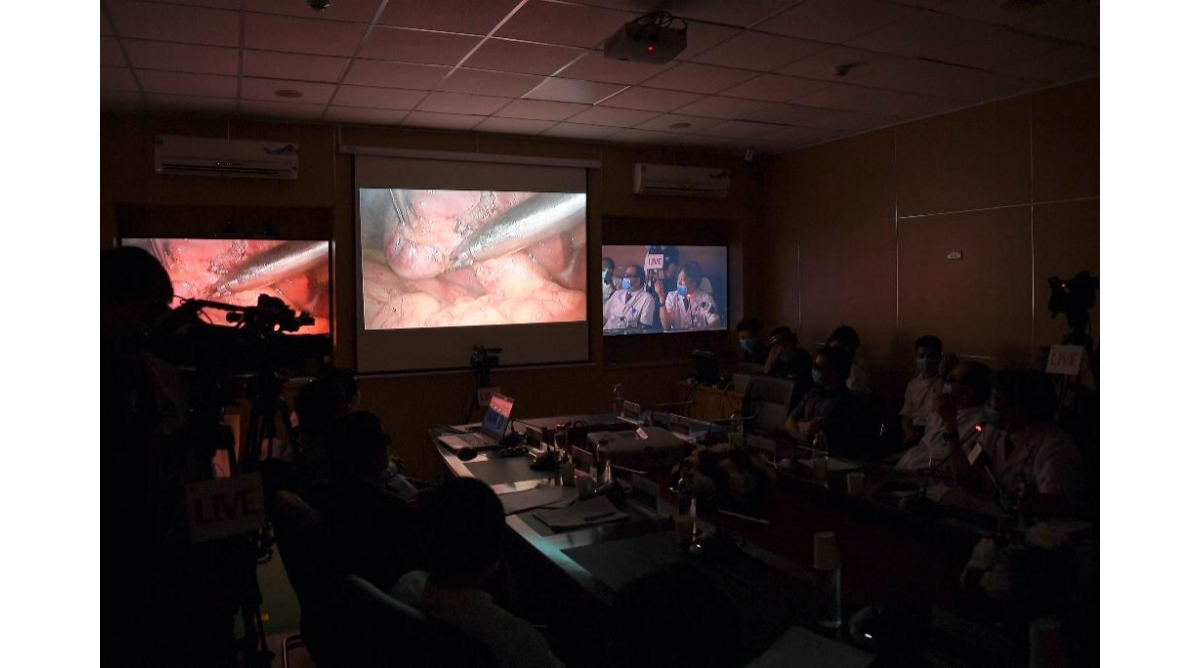
Telesurgery consultation via the web-based platform.

Another breakthrough of telemedicine in PGH was successfully applying telemedicine in surgery. For example, on August 6, 2020, PGH organized a telemedicine cardiovascular surgery program for PGH and Hanoi Heart Hospital for the case of a 55-month-old child with a ventricular septal defect hole under 2 aortas (Figure 1). Before the operation, surgeons and technicians had a teleconsultation with cardiologists from Hanoi Heart Hospital to plan the surgery strategy. During the operation, a camera livestreamed the operation. The surgeons received advice and guidance from the cardiologists during the operation. Furthermore, doctors at Hanoi Heart Hospital have successfully carried out the first web-based heart surgeries in Vietnam for patients at PGH via the telehealth system that was developed by Viettel Group (Figure 2). Currently, the hospital has conducted 10 heart operations, including treatments for mitral stenosis, mitral regurgitation, ventricular septal defects, aortic valve stenosis, mucous tumors, atrial fibrillation, and heart failure.

## Telehealth Has Changed the Way That Doctors From PGH and National Hospitals Collaborate During the COVID-19 Pandemic

The COVID-19 outbreak has changed the way that people contact and communicate with PGH and national hospitals. Telehealth is a model that uses electronic information and telecommunication technologies to support and promote long-distance clinical health care, patient and professional health–related education, public health care facilities, and health administration agencies. Telehealth technology helps doctors connect patients with physicians through video calls, emails, and web-based patient portals and enables real-time consultations between specialists. A quick web-based visit can improve diagnosis and treatment efficiency, help with improving patients’ experiences, and reduce the number of complications and hospital admissions. Recently, many researchers have indicated that using telehealth for specialty visits connects primary care physicians, specialists, and patients, resulting in the enhanced coordination of care and speedy diagnoses [15]. This service is also comfortable and beneficial for patients who need follow-up care without a physical exam for monitoring medication side effects or patients who have nonurgent questions after surgery [12].

Telemedicine has changed the approach of health care services and how they are delivered. Telemedicine has also offered many advantages for doctors and patients in PGH, as shown in the following sections.

## Strengthening the Quality of Health Care Services in Primary Health Care Centers and Remote Communities

Doctors and physicians in remote, rural areas of Phutho province can learn from experienced doctors. This is an opportunity for PGH to receive professional support remotely and gradually improve the quality of medical services. Often, using smartphones to transmit images and videos is much more convenient than using formal videoconferencing technology and is often preferred by doctors [16].

Patients can easily receive consultations and prescriptions from leading physicians in national hospitals without having to travel long distances, which saves time and money. From a psychological perspective, rural patients in Vietnam always want to be examined and cared for by central medical staff who are believed to have more experience, knowledge, and skills than lower-level medical teams. Therefore, telemedicine increases patients’ access to care when it involves extended specialist and physician access. This results in telemedicine improving patient engagement and satisfaction.

## Telemedicine Can Effectively Slow Down the Spread of Infection

Telehealth is an effective option for supporting the fight against the outbreak of COVID-19, as it reduces the risk of coming into contact with people with SARS-CoV-2 infection [17]. Hospital-acquired infection is a serious problem in Vietnam—a tropical country that has a high risk of nosocomial infection. Telemedicine has the advantage of digital health care solutions that can prevent the spread of the pandemic nationwide because they help reduce the amount of direct contact with patients and decrease the risk of infection for health staff. Thus, the benefits of telemedicine are obvious when it comes to social distancing and decreasing the spread of diseases.

## Limitations

Telehealth and telemedicine have emerged as new models of health technology and have been applied in many hospitals in Vietnam during the COVID-19 outbreak. However, there are some limitations.

First, telemedicine may not suit every person or situation. Second, medical data maybe at risk of being violated by hackers and being accessed by other criminals, especially if a patient accesses telemedicine services on a public network or via an unencrypted channel. Third, care may be delayed when a person needs emergency care, as accessing telemedicine services first may delay treatment, particularly since a doctor cannot provide lifesaving care or conduct laboratory tests digitally. Fourth, technological concerns can be challenging; a weak internet connection can make it especially difficult to offer quality care. Fifth, not all hospitals are equipped for telemedicine, particularly those that lack computer terminals, which are necessary for implementing telemedicine services.

## Data Availability

The data reported in this paper can be made available by the corresponding author upon request from qualified investigators.

## Conclusion

Telehealth has emerged as a model of modern technology for health care services in Vietnam during the COVID-19 outbreak. This report is the first to provide the early results of counseling and support activities of remote examination and treatment activities in Vietnam. It is expected that these remote examination and treatment consultancy activities, which are based on an information technology platform, will increasingly promote and ensure the efficiency and sustainability of telehealth in Vietnam.

The results from PGH show the advantages of using telehealth in remote examinations that adhere to the treatment system standards of the Ministry of Health. By integrating modern data transmission technology and using high-speed internet, the system is capable processing data in real time and supporting the remote delivery of surgery. Doctors at higher-level hospitals can directly guide surgeons of lower-level hospitals, thereby shortening the process of treating patients in emergencies.

The COVID-19 pandemic has allowed unprecedented opportunities for telemedicine to develop. These opportunities require heightened engagement from the government to make sure that a regulatory foundation is implemented. A synchronized telemedicine system should be built in Vietnam in the future.

## Abbreviations

PGH: Phutho General Hospital
QD-BYT: Quyet dinh-Bo y te
